# Statistical design and optimization of single cell oil production from sugarcane bagasse hydrolysate by an oleaginous yeast *Rhodotorula* sp. IIP-33 using response surface methodology

**DOI:** 10.1186/2193-1801-3-691

**Published:** 2014-11-25

**Authors:** Sheetal Bandhu, Diptarka Dasgupta, Jawed Akhter, Pankaj Kanaujia, Sunil K Suman, Deepti Agrawal, Savita Kaul, Dilip K Adhikari, Debashish Ghosh

**Affiliations:** Biofuels Division, CSIR-Indian Institute of Petroleum, Mohkampur, Dehradun, 248 005 Uttarakhand India; Analytical Sciences Division, CSIR-Indian Institute of Petroleum, Mohkampur, Dehradun, 248 005 Uttarakhand India

**Keywords:** Lignocellulosic biomass, Pentose sugar, Yeast lipid, *Rhodotorula* sp, Response surface methodology

## Abstract

Single cell oil production from sugarcane bagasse hydrolysate by oleaginous yeast Rhodotorula sp. IIP-33 was analyzed using a two stage statistical design approach based on Response Surface Methodology. Variables like pentose sugar, (NH_4_)_2_SO_4_, KH_2_PO_4_, yeast extract, pH and temperature were found to influence lipid production significantly. Under optimized condition in a shake flask, yield of lipid was 2.1199 g with fat coefficient of 7.09 which also resembled ~99% similarity to model predicted lipid production. In this paper we are presenting optimized results for production of non polar lipid which could be later deoxygenated into hydrocarbon. A qualitative analyses of selective lipid samples yielded a varying distribution of free acid ranging from C_6_ to C_18_, majoring C_16:0_, C_18:0_ and C_18:1_ under different fermentation conditions.

## Introduction

Plant and algae based natural fatty oils find industrial applications with continuous increasing demand as oleochemicals and biofuel feedstock. Transesterification of this lipid to its corresponding esters yield a diesel substitute which lacks in drop in’ characteristics like hydrocarbon based fossil fuel (Wackett 2008). Instead, selective de-oxygenation (hydrotreatment) would yield renewable hydrocarbon of desired fuel range (gasoline, aviation jet and diesel) (Verma et al. 2011). However, plant derived lipid can hardly meet our future energy demand irrespective of transesterification or hydrotreatment. Apart from various oil seed bearing plants and microalgae, microbial lipids offer some potential advantages due to their short generation time (80 h with respect to 24 months for plants or 2 months for algae); limited space requirement (could avoid food fuel debate); generation of uniform lipid fractions (irrespective of climate and country) (Li et al. 2008). However, high processing cost still imposes a challenge for its commercialization. Maximum lipid accumulation by oleaginous microorganisms using cheap carbon sources like lignocellulosic biomass derived fermentable sugars and further recovery of single cell oil are undoubtedly major challenges for its commercial success (da Silva et al. 2014; Flores et al. 2000). Sugarcane bagasse can be a potential biomass source in terms of fermentable sugars (~60% holocellulose content) with an average production of 350 MMT (million metric tonne) per annum. Indian sugar mills receive 40% of the total sugarcane produced. 50-55% is crushed in unorganized sector and 8-10% is utilized as seed for future crop. Normally 30% bagasse is obtained from total cane crushed in a sugar mill. Out of total bagasse generated in sugar mill, 75-85% is used to generate boiler steam and rest 15-25% is surplus for other uses mainly papermaking and co-generation. Thus Indian sugar mills generate ~40-42 MMT bagasse which can be effectively hydrolyzed and sachcharified to extract fermentable sugars for valorization to fuels or specialty chemicals instead of boiler steam generation (Jain et al. 2011). Oleaginous microorganisms accumulate lipid when intracellular AMP concentration declines due to depletion of culture nitrogen concentration. Hence microbial biomass generated under carbon limiting condition, channelize their carbon flux for lypogenesis during nitrogen limiting condition, in presence of high sugar density (Botham and Ratledge 1979). Flux is further driven by the reductant NADPH generated during formation of pyruvate from oxaloacetate via malate (Ratledge 2004). Temperature induced changes are reported for fatty acid and lipid quantity and composition in many oleaginous microorganisms. *Rhodotorula* sp. IIP-33 (hence forth mentioned as IIP-33) is one such yeast and its growth and lipid accumulation characteristics have been reported (Saxena et al. 1998).

One of the unique characteristics of IIP-33 is its ability in utilizing both pentose and hexose sugar for cell biomass generation and lipid accumulation (Chandra 1997). Cell biomass was grown with pentose rich fractions obtained after acid and steam hydrolysis of sugarcane bagasse (SCB) and nitrogen limiting conditions were obtained by adding concentrated pentose stream of SCB hydrolysate. In this paper, we have targeted quantitative accumulation (weight basis) of non polar lipid by IIP-33 by RSM (Response Surface Methodology) via two step approach. Initial screening was performed with Plackett-Burman Design (PBD) (Plackett & Burman 1946) method to identify crucial parameters affecting lipid yield and to the degree based on their individual effect and interactions through Box-Behnken Design (BBD) (Box & Behnken 1960). Further, we had selected 13 lipid samples with varying weights from three different temperatures varying in (carbon/nitrogen) C/N ratios for qualitative analyses of lipid through Gas chromatography coupled with mass spectroscopy (GC/MS) to find any compositional variation in terms of free fatty acids.

## Materials and methods

### Materials

Sugar cane bagasse was procured from local sugar mill in Doiwala, Dehradun, India for hydrolysis. SCB was pretreated with steam and 4% w/w H_2_SO_4_ in 1:10 solid–liquid ratio for 90 minutes holding time at 120°C temperature and 4 bar pressure to extract pentose rich fraction. The broth was neutralized by over-liming. Pentose sugar was used as carbon source for cell biomass generation. Pentose stream was further concentrated and used in all experimental shake flasks with desired sugar concentrations as per experimental design (Tables 1 and 2).

**Table 1:** **Plackett burman design for initial screening of factors**

Run order	Std. order	A	B	C	D	E	F	G	H	I	Response variable: Lipid wt.	
		Xylose	(NH _4_ ) _2_ SO _4_	Na _2_ HPO _4_	KH _2_ PO _4_	Yeast extract	MgSO _4_	Time	pH	Temp	Experimental	Predicted
		g	g	g	g	g	g	h	-	°C	g	g
1	7	+1	-1	-1	-1	+1	-1	+1	+1	-1	1.4363	1.4482
2	8	+1	+1	-1	-1	-1	+1	-1	+1	+1	1.0892	1.089
3	9	+1	+1	+1	-1	-1	-1	+1	-1	+1	0.7101	0.710
5	10	-1	+1	+1	+1	-1	-1	-1	+1	-1	1.3764	1.3883
6	6	-1	-1	-1	+1	-1	+1	+1	-1	+1	1.7299	1.7418
7	1	+1	+1	-1	+1	+1	+1	-1	-1	-1	0.5932	0.5932
8	5	-1	-1	+1	-1	+1	+1	-1	+1	+1	1.0931	1.0931
9	4	-1	+1	-1	+1	+1	-1	+1	+1	+1	1.4091	1.3972
10	3	+1	-1	+1	+1	-1	+1	+1	+1	-1	2.6138	2.6019
11	12	-1	-1	-1	-1	-1	-1	-1	-1	-1	0.5911	0.5792
12	11	+1	-1	+1	+1	+1	-1	-1	-1	+1	1.8509	1.8509

**Table 2:** **Box Behnken design for lipid production**

Std order	Run order	A	B	C	D	E	F	Lipid weight	Model predicted response
		Xylose	(NH _4_ ) _2_ SO _4_	KH _2_ PO _4_	Yeast extract	pH	Temperature		
		g	g	g	g	-	°C	g	g
32	1	0	-1	0	0	+1	-1	1.492	1.4913
45	2	-1	0	-1	0	0	-1	0.776	0.779
42	3	0	+1	0	0	-1	-1	1.177	1.1767
10	4	0	0	+1	+1	0	-1	1.465	1.4742
17	5	0	-1	0	0	-1	-1	1.253	1.252
24	6	0	0	-1	-1	0	-1	1.206	1.205
25	7	0	0	-1	+1	0	-1	1.326	1.3231
12	8	+1	0	-1	0	0	-1	1.752	1.7539
38	9	0	0	+1	-1	0	-1	1.364	1.3654
9	10	0	+1	0	0	+1	-1	1.410	1.4097
27	11	1	0	+1	0	0	-1	1.972	1.9689
6	12	-1	0	+1	0	0	-1	0.878	0.8754
4	13	0	-1	-1	0	-1	0	1.226	1,2276
20	14	0	+1	+1	0	+1	0	1.545	1.5445
28	15	+1	+1	0	+1	0	0	1.938	1.9378
1	16	-1	-1	0	-1	0	0	0.844	0.8457
14	17	+1	-1	0	-1	0	0	1.872	1.8731
44	18	-1	0	0	-1	+1	0	0.868	0.8691
36	19	0	+1	-1	0	+1	0	1.380	1.381
39	20	-1	0	0	+1	-1	0	0.892	0.8917
35	21	0	+1	-1	0	-1	0	1.153	1.1542
34	22	+1	0	0	-1	-1	0	1.683	1.6848
37	23	-1	+1	0	-1	0	0	0.746	0.7466
16	24	+1	0	0	+1	+1	0	2.09	2.065
18	25	-1	0	0	-1	-1	0	0.741	0.742
31	26	-1	+1	0	+1	0	0	0.899	0.8989
26	27	+1	0	0	-1	+1	0	2.024	2.0251
43	28	0	-1	-1	0	+1	0	1.459	1.4607
52	29	0	+1	+1	0	-1	0	1.307	1.3061
7	30	0	-1	+1	0	-1	0	1.380	1.3795
30	31	+1	-1	0	+1	0	0	1.992	1.9918
3	32	0	-1	+1	0	1	0	1.624	1.6243
29	33	+1	+1	0	-1	0	0	1.817	1.818
5	34	-1	0	0	+1	+1	0	1.023	1.0229
13	35	-1	-1	0	+1	0	0	0.997	0.9969
41	36	+1	0	0	+1	-1	0	1.802	1.8019
40	37	0	+1	0	0	+1	+1	1.514	1.5157
11	38	+1	0	+1	0	0	+1	2.0743	2.0744
2	39	-1	0	+1	0	0	+1	0.981	0.9829
21	40	0	+1	0	0	-1	+1	1.282	1.2834
8	41	-1	0	-1	0	0	+1	0.882	0.8825
19	42	0	0	-1	-1	0	+1	1.291	1.2855
46	43	0	-1	0	0	+1	+1	1.593	1.5935
22	44	0	0	+1	+1	0	+1	1.604	1.6027
15	45	+1	0	-1	0	0	+1	1.854	1.8554
23	46	0	0	-1	+1	0	+1	1.445	1.4476
33	47	0	-1	0	0	-1	+1	1.354	1.355

### Microorganism and culture conditions

IIP-33 (MTCC 2518), an oleaginous yeast (GenBank Accession number KF313359) was isolated from mineral oil contaminated local soil as reported by Saxena et al. (1998). Optimally grown IIP-33 at 32°C and pH 4.5 was used in this experimental study. Stock culture was maintained on YPX agar medium (composition in g/L; yeast extract, 10.0; peptone, 20.0; xylose 20.0; *agar agar* 20.0; pH 4.5-5.0.

### Experimental design

Biomass generation was carried out in SCB hydrolysate (20 g.L^-1^) with peptone (20 g.L^-1^) and yeast extract (10 g.L^-1^) in 10 L fermenter (INCELTECH LH Series 210 fermenter, Berkshire, England) at 32°C. Growth was terminated after 90% consumption of sugar which was nearly 12 h from the onset of inoculation. Nutrient screening and optimization for lipid production with generated biomass were performed in shake flasks (0.5 L working volume; 20 g cell on dry basis) in biomass hydrolysate with various nutrients according to experimental design. Physical parameters, temperature, pH and fermentation time were maintained as per experimental design (Table 1). Experiments were carried out in duplicate sets and final data were reported in terms of mean values. Experimental design and statistical analysis were performed with *Reliasoft* Design of Experiment (DOE) software with a risk factor (α) of 0.05 (i.e. 95% level of confidence) for both PBD and BBD. Coefficient of regression (R^2^adj) with value over 0.98 was selected as criterion for acceptance of predicted model. Variables with P values lower than 0.05 were considered to have a significant effect on lipid production.

### Plackett Burman design for initial screening

A two level PBD experimental matrix was set up to identify factors and estimate their significance in lipid production by IIP-33. A total of nine independent variables were selected for this study; physical parameters such as temperature, pH, fermentation time and media components such as xylose concentration, ammonium sulphate [(NH_4_)_2_SO_4_], disodium hydrogen phosphate [Na_2_HPO_4_] and potassium di-hydrogen phosphate [KH_2_PO_4_], yeast extract and magnesium sulphate [MgSO_4_]. Each variable was represented at three levels, low (-1), medium (0) and high (1) concentration (Table 3). According to PBD, eleven trials were performed with lipid content (Y) as response (Table 1). Final predicted model was linear, with only main effects in consideration.

**Table 3:** **Variables with their coded levels**

Serial number	Variables	Low	Medium	High
		(-1)	(0)	(+1)
1	Xylose (g)	10	20	30
2	(NH_4_)_2_SO_4_ (g)	0.5	0.75	1
3	Na_2_HPO_4_ (g)	0.188	0.2815	0.375
4	(KH_2_PO_4_) (g)	0.35	0.525	0.70
5	Yeast extract (g)	0.25	0.50	0.75
6	MgSO_4_ (g)	0.175	0.3375	0.50
7	Time (h)	18	24	30
8	pH	4	5	6
9	Temp (°C)	30	34	38

1

Response indicated dependent variable in terms of overall lipid production (g.L^-1^), a being model intercept. X_i_ represented different levels of independent variables with b_i_ coefficients as predicted by the Equation 1.

### BBD design for lipid optimization

Following Placket Burman screening of factors, BBD was applied to further develop mathematical correlations between key independent variables on lipid production. BBD matrix was constructed with six significant factors (xylose concentration, (NH_4_)_2_SO_4_, KH_2_PO_4_, yeast extract, pH and temperature) each having 3 levels (-1, 0 and 1) with 47 experimental designs as shown in Table 2. All non-significant factors predicted by PBD like time, MgSO_4_ and Na_2_HPO_4_, were kept at their respective low level values (Table 3). BBD response was fit by a second-order polynomial in order to correlate response with independent factors. ANOVA analysis of predicted model was carried out to evaluate its statistical significance. System response predicted by second order polynomial was represented as a combination of linear, interaction and quadratic effect of independent variables on system response, either + ve or –ve.
2

Where, x_i_ represented independent variables, β_0_ was intercept, β_i_, linear term coefficients, β_ij_ indicated interaction terms and β_ii_ represented quadratic effect terms.

### Model validation in shake flask

BBD study predicted optimized condition for lipid production in terms of key independent variables having significant impact on lipid production. A shake flask study under optimized condition was performed to validate correctness of the predicted computational model.

### Qualitative estimation of lipid for selected samples

Amongst 47 software predicted experimental sets 12 lipid samples were selected for qualitative distribution of fatty acid in terms of carbon numbers through GC/MS. Samples were selected on the basis of maximum quantitative lipid yield under three different temperatures with varying C/N ratio, temperature and pH for lipid fermentation and relative fatty acids distribution was tabulated (Table 4). Finally, this was also compared with the lipid generated in model validated flask.

**Table 4:** **Relative fatty acid of 13 selective lipid samples**

Sl. No.	Temperature	C/N	Lipid	pH	Flask	C _5_ sugar ^δ^	Fat coefficient ^ψ^	≤ C _6_ to ≥ C _14_	C _16:0_	C _16:1_	C _18:0_	C _18:1_	C _18:2_	C _18:3_
#	°C	~	g	-	#	g	%	relative presence of fatty acids of corresponding carbon numbers						
1	30	25	0.8780	5	12	10	8.78	++	+	-	+	+	-	+
2	30	50	1.4650	5	4	20	7.33	+	++	+	+	+	-	+
3	30	75	1.9720	5	11	30	6.57	++	++	+	+	++	-	+
4	34	25	0.8990	5	26	10	8.99	+	+	-	-	+	+	-
5	34	50	1.9380	5	15	30	6.46	+	++	+	+	++	++	-
6	34	75	2.0243	6	27	10	20.24^η^	+	++	+	++	++	++	-
7	34	75	2.0900	6	24	30	6.96	++	++	+	++	++	++	-
8	34	100	1.8720	5	17	30	6.24	+	++	-	++	++	++	-
9	34	100	1.9920	5	31	30	6.64	+	++	+	++	++	++	-
10	38	25	0.8820	5	42	10	8.82	++	+	+	+	+	+	-
11	38	50	1.5140	6	38	20	7.57	+	+	+	+	+	++	-
12	38	75	2.0740	5	39	30	6.91	++	++	-	+	++	++	-
**13**	**38**	**100**	**2.1199**	**6**	**model**	**30**	**7.06** ^*^	**++**	**++**	**+**	**++**	**++**	**++**	**-**

+ indicates presence of fatty acids with corresponding carbon numbers as per GC/MS; ++ indicates presence of corresponding fatty acids in higher quantity as per GC/MS; δ gram of pentose sugar (C_5_) present in prehydrolysate as carbon source in all flasks; ψ (yielded lipid/consumed sugar) × 100 = fat coefficient (%); η maximum fat coefficient due to low carbon content and high C/N ratio (very low nitrogen content); not considered as model data; * fat coefficient as per validated model.

### Estimation of lipid and analytical methods

After fermentation, cells were separated by centrifugation and dried. Lipid was extracted from dry cell biomass in two stage solvent extraction method. In first stage, total lipids were extracted with 1:3 chloroform/methanol (CHCl_3_:CH_3_OH) and then followed by n-hexane. Solvents were evaporated under vacuum to collect and quantify lipid on weight basis. Estimation of sugar (xylose in prehydrolysate) during cell biomass generation and lipid maturation was quantified by High pressure liquid chromatography (HPLC) (UFLC, Shimadzu, Japan) with PL Hiplex-H acid 8 μm column (100 × 7.7 mm diameter, by PL Polymer laboratory, UK). Column was eluted with a mobile phase 1 mM sulfuric acid at a flow rate of 0.7 ml.min^-1^ at column oven temperature 70°C with refractive Index (RI) detector. Total nitrogen was analyzed by UV method as per ASTM D 4629 with Total Nitrogen Analyzer (TN3000, Thermo-Fischer). Known quantity of cellular broth (1 ml) was used for determination of dry cell weight (DCW) by hot air drying of cell pellets in microfuges. Average of triplicate data was considered for DCW determination. Qualitative gas GC/MS analyses were performed with a Hewlett Packard 5890 gas chromatograph equipped with a model 5972 mass selective detector (Hewlett Packard, USA). A SGE BPX5 capillary column with 30 m length × 0.25 mm internal diameter × 0.32 μm film thickness was used. GC oven temperature was programmed from 50°C (hold for 2 min) to a final temperature of 300°C at 10°C.min^-1^ (hold for 30 min). Helium was used as a carrier gas under constant flow (1.2 ml.min^-1^) mode. Transfer line temperature was fixed to 280°C. All samples were analyzed in splitless mode at an injection temperature of 250°C. 1 μL injection of samples was performed with a 5 μL micro syringe. Mass spectrometric analyses were carried out in electron ionization (EI) mode with 70 eV ionization energy and 150°C quadruple temperature. Ion source temperature was kept at 230°C. Mass spectra for all target compounds were acquired in full scan mode after derivatization with BSTFA (N, O-bis-trimethylsilyl trifluoroacetamide, Sigma Aldrich, India). Derivatization was performed by taking 50 μL analyte solutions in hexane (approximate concentration of 1 mg.ml^-1^) and adding 100 μL of BSTFA in a glass vial with screw cap and Teflon seal. This vial was kept at 80°C for 1 hour and injected in GC/MS.

## Results and discussion

### Screening of key variables affecting lipid production

Optimum microbial lipid production required a perfect association of micro and macro nutrients such as carbon and nitrogen along with other physical parameters namely temperature and pH whereby a suitable environment was provided for yeast growth and proliferation. A medium with excess pentose sugar in the form of SCB hydrolysate and limited nitrogen content greatly enhanced lipid production. Oleaginous profile was significantly affected by C/N molar ratio of the culture (Ratledge and Wynn 2002) with higher values (C/N 50) of the same being more favourable for lipid production (Braunwald et al. 2013). However, this resulted in reduced biomass yield and specific growth rate of IIP-33. Temperature played a significant role in determining lipid quality of the yeast. Slight variation in temperature altered fatty acid composition of the lipids. As culture temperature increased, relative content of unsaturated fatty acids in cellular lipids increased (Hamid and Khan 1991; Amaretti et al. 2010). This is attributed to better cellular adaptation at those temperatures. Thus by fine tuning temperature, lipid quality could be significantly altered. Inorganic salts such as Na_2_HPO_4_ and KH_2_PO_4_ acted as buffering agents and helped to maintain cell integrity during growth and also played a significant role in phospholipid accumulation. Magnesium ions were important cofactors and its enhanced levels had significant influence on cell growth and lipid accumulation. Yeast extract was a rich source of vitamins and promoted cell growth and proliferation (Dasgupta et al. 2013). Elevated levels of magnesium enhanced accumulation of lipid by promoting acetyl CoA carboxylase enzyme activity (Janβen et al. 2013). pH primarily affected lipid content and lipid quality. Lipid content generally increased when pH is kept at higher than optimal. Low pH resulted in accumulation of higher saturated fatty acids which reducd membrane fluidity.

**Figure 1:**
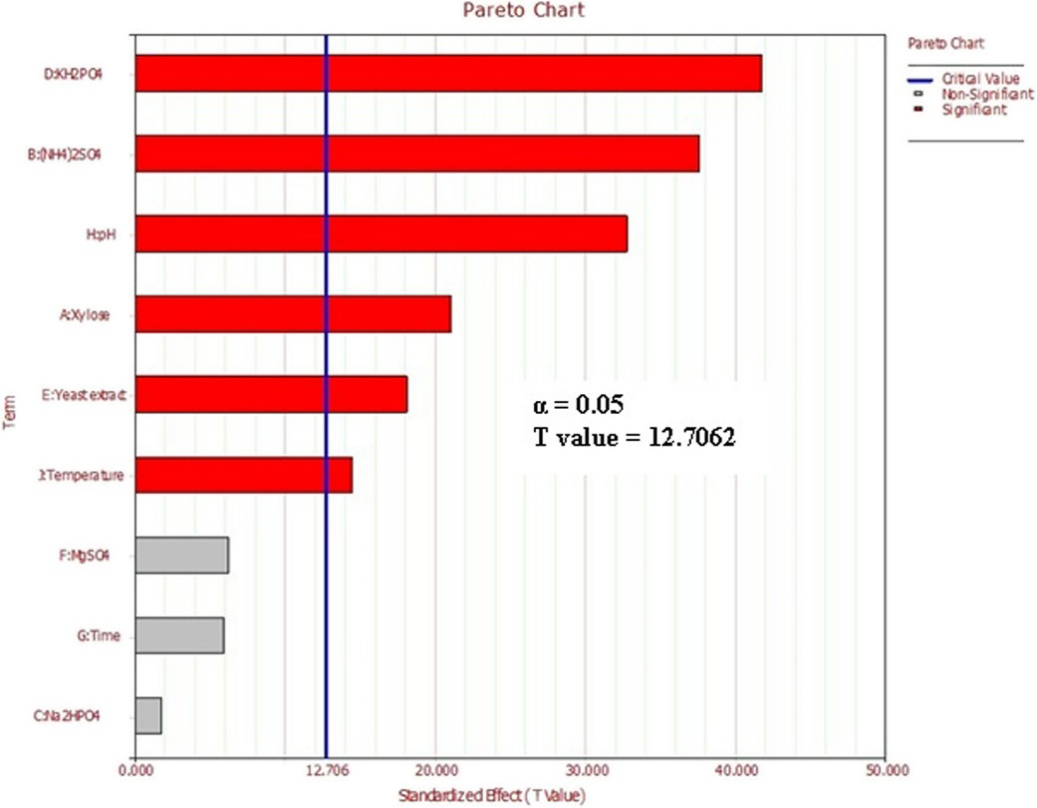
**Pareto chart for Placket Burman screening of variables.**

Microbial lipid production by oleaginous yeast was quantified in terms of both total lipid content and lipid coefficient (Holdsworth and Ratledge 1988). Lipid content referred to production yield, whereas lipid coefficient was related to its efficiency of bioconversion from substrate to lipid. Table 1 summarized lipid contents obtained from Plackett–Burman experimental design for 11 trials with two levels of concentration for each independent variable. Pareto chart analysis (Figure 1) identified key variables for lipid production based on PBD experimental study. 6 factors among selected ones such as KH_2_PO_4_, (NH4)_2_SO_4_, pH, Xylose concentration, Yeast extract and temperature with T values above threshold (12.706 in this study) and P values lower than 0.05 were found to have a significant effect on the system response. Regression data table analysis for PBD (Table 5) highlighted that the components MgSO_4_, fermentation time and Na_2_HPO_4_ had no significant effect on system response as their P values were above the selected criteria for 95% level of confidence. Positive sign (+) for the effective component suggested that, further optimization preferred a similar or higher value than indicated one, and vice versa. The model considering variable main effects was highly accurate with R^2^_adj_ value of 0.99 with experimental and model predicted responses was near identical. 6 independent variables having significant effect on system response were further evaluated using BBD with 3 levels of variation while rest non-significant factors were kept at their lowermost values.

**Table 5:** **Regression analysis for PBD**

Term	Effect	Coefficient	Standard Error	T Value	P Value
**Intercept**		**2.2278**	**0.051**	**43.7165**	**0.0146**
**A:Xylose**	**0.4396**	**0.2198**	**0.0103**	**21.328**	**0.0298**
**B:(NH**_**4**_ **)**_**2**_ **SO**_**4**_	**-0.7801**	**-0.3901**	**0.0103**	**-37.85**	**0.0168**
C:Na_2_HPO_4_	0.0842	0.0421	0.0207	2.0361	0.2906
**D:KH**_**2**_ **PO**_**4**_	**1.7324**	**0.8662**	**0.0206**	**42.0253**	**0.0151**
**E:Yeast extract**	**-0.7572**	**-0.3786**	**0.0206**	**-18.369**	**0.0346**
F:MgSO_4_	-0.2661	-0.1331	0.0206	-6.456	0.0978
G:Time	0.1269	0.0635	0.0103	6.1584	0.1025
**H:pH**	**0.6811**	**0.3405**	**0.0103**	**33.0432**	**0.0193**
**J:Temperature**	**0.3025**	**0.1513**	**0.0103**	**14.678**	**0.0433**

*Significant variables have been indicated in bold.

### Optimization of medium components and physical factors by Box–Behnken factorial design

Lipid production via BBD matrix is shown in Table 2. ANOVA calculations illustrated in Table 6 depicted that the model F and P values were 2.61 × 10^4^ and 2.93 × 10^-40^. Hence model was significant with 95% level of confidence with linear, interaction and exhibited quadratic effects. Coefficient of determination of the predicted model (R_adj_2) was calculated as 0.99. Statistical equation was unable to explain only 1% variability in the response data. Response values obtained with individual runs were near identical to model predicted data values. This indicated a good agreement between experimental and predicted values for lipid content (Table 7). T-value measured how large a coefficient was in relationship to its standard error (i.e. a ‘signal-to-noise’ type). It was observed that main effects were significant for each of the six coded factors whereas interactions among xylose and (NH_4_)_2_SO_4_, xylose and KH_2_PO_4_, xylose and pH, yeast extract and temperature etc. were important as indicated by their high T and low P values. The final response i.e. lipid content modelled as a function of independent variables in terms of their coded values with both main and interaction affected in consideration has been shown below as:

**Table 6:** **ANOVA data table for BBD**

Source of Variation	Degrees of freedom	Sum of squares [Partial]	Mean Squares [Partial]	F Ratio	P Value
**Model**	27	7.1309	0.2641	**2.61E + 04**	**2.93E-40**
**Linear Effects**	6	7.091	1.1818	**1.17E + 05**	**2.37E-44**
**Interaction Effects**	15	0.0329	0.0022	**216.6106**	**8.93E-19**
**Quadratic Effects**	6	0.0047	0.0008	**77.903**	**8.28E-13**
Residual	20	0.0002	1.01E-05		
Lack of Fit	20	0.0002	1.01E-05		
Total	47	7.1311			

*Significant model effects have been indicated in bold.

**Table 7:** **Significance of term coefficients for BBD**

Term	Coefficient	Standard error	T value	P value
**Intercept**	**1.3661**	0.0026	517.7999	**2.73E-07**
**A:Xylose**	**0.5166**	0.0006	795.4539	**1.78E-15**
**B:(NH**_**4**_ **)**_**2**_ **SO**_**4**_	**-0.0383**	0.0006	-58.9734	**0.0011**
**C:KH**_**2**_ **PO**_**4**_	**0.0789**	0.0007	116.0206	**1.37E-05**
**D:Yeast extract**	**-0.0181**	0.0007	99.6837	**0.0477**
**E:pH**	**0.1179**	0.0006	181.5286	**1.16E-06**
**F:Temperature**	**0.0522**	0.0007	76.8728	**3.53E-07**
**AB**	**0.011**	0.0011	9.7794	**4.60E-09**
**AC**	**0.0297**	0.0011	26.3607	**0**
**AD**	**-0.0081**	0.0008	-10.2154	**2.21E-09**
**AE**	**0.0533**	0.0011	47.4151	**0**
AF	-5.00E-04	1.10E-03	-0.451	0.6568
BC	0	0.0011	0	1
BD	0.0003	0.0011	0.2355	0.8162
BE	-0.0016	0.0008	-1.9723	0.0626
BF	9.00E-04	1.10E-03	0.8353	0.4134
CD	-0.0023	0.0013	-1.8153	0.0845
**CE**	**0.0029**	0.0011	2.593	**0.0174**
CF	1.00E-03	9.00E-04	1.1744	0.254
DE	0.001	0.0011	0.9209	0.3681
**DF**	**0.011**	1.30E-03	8.6233	**3.59E-08**
EF	-2.00E-04	1.10E-03	-0.1742	0.8634
**AA**	**0.0124**	0.0013	9.2964	**1.06E-08**
BB	8.72E-05	0.0014	0.0637	9.50E-01
**CC**	**0.009**	0.0015	5.9536	**8.02E-06**
**DD**	**0.01**	0.0013	7.6217	**2.44E-07**
**EE**	**9.50E-03**	1.10E-03	8.2952	**6.63E-08**
**FF**	**9.00E-03**	6.00E-04	15.4471	**1.40E-12**

*Significant variables have been indicated in bold.

**Figure 2:**
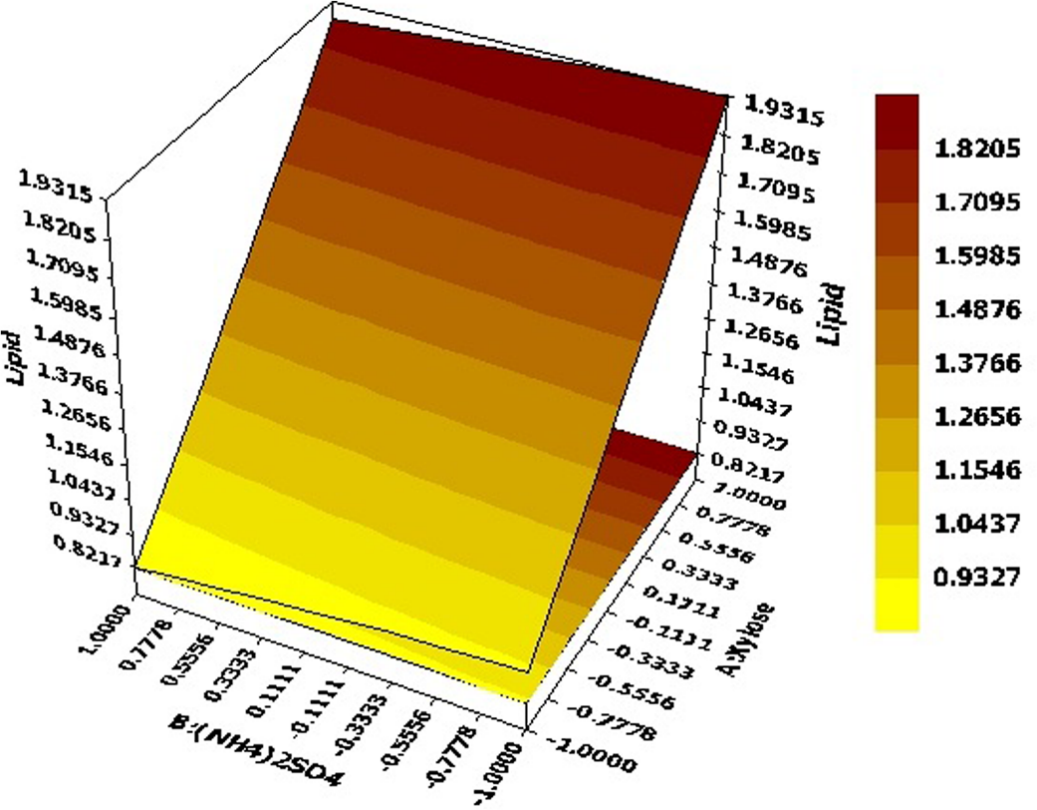
**Effects of Xylose and (NH**_**4**_ **)**_**2**_ **SO**_**4**_ **conc. on lipid production; Hold values C =0 (KH**_**2**_ **PO**_**4**_ **= 0.525 g), D =0 (Yeast extract = 0.5 g), E =0 (pH = 5), F =0 (Temperature = 34°C).**

**Figure 3:**
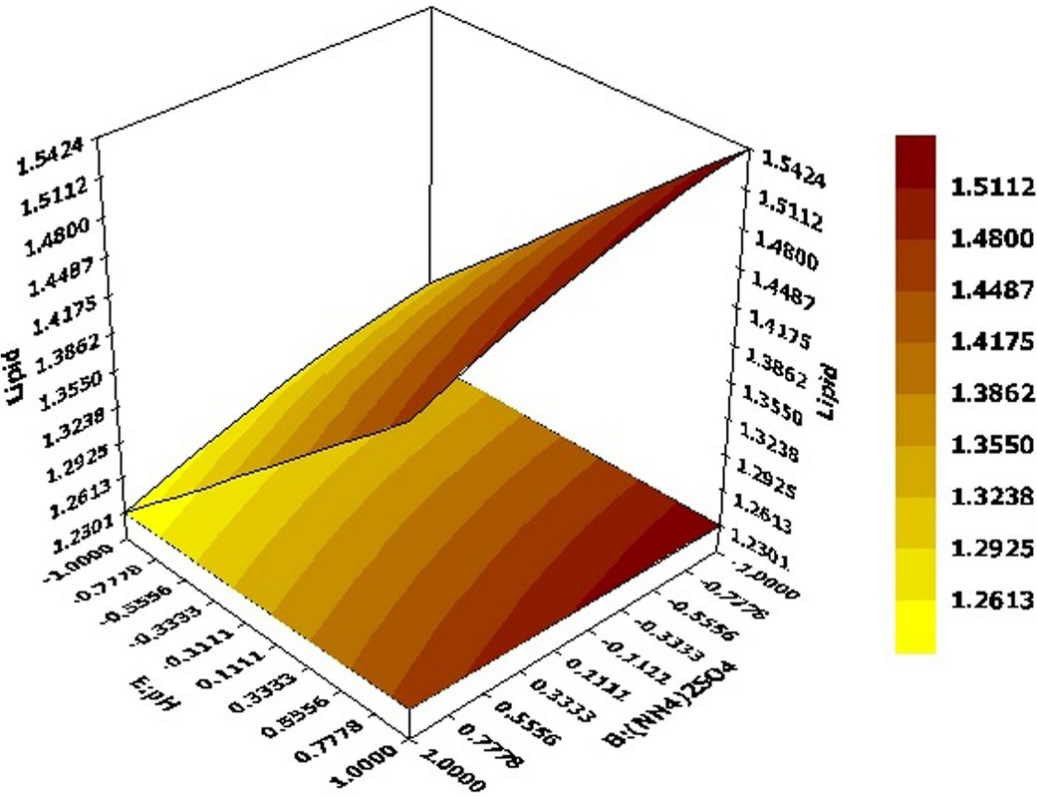
**Effects of (NH**_**4**_ **)**_**2**_ **SO**_**4**_ **conc. and pH on lipid production; Hold values A =0 (Xylose conc. =20 g), C =0 (KH**_**2**_ **PO**_**4**_ **= 0.525 g), D =0 (Yeast extract = 0.5 g), F =0 (Temperature = 34°C).**

**Figure 4:**
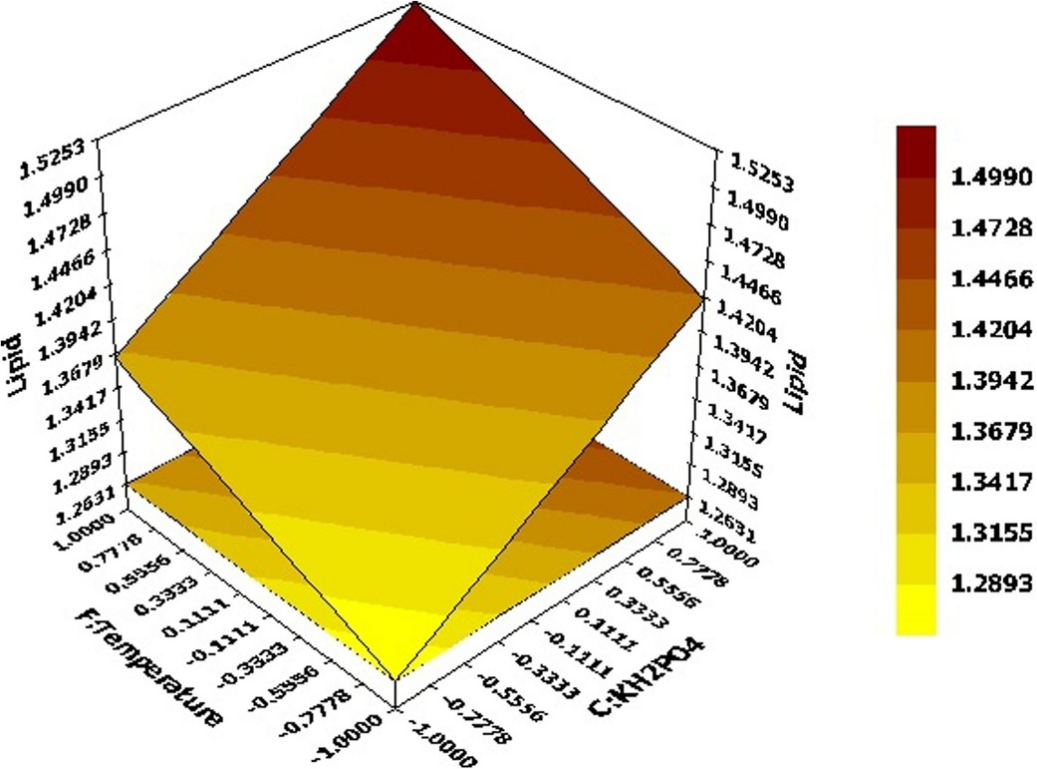
**Effects of KH**_**2**_ **PO**_**4**_ **and Temperature on lipid production; Hold values A =0 (Xylose =20 g), D =0 (Yeast extract = 0.5 g), E =0 (pH = 5), B =0 ((NH**_**4**_ **)**_**2**_ **SO**_**4**_ **= 0.75 g).**

3D response surface graphs displayed characteristic effects of key process variables on lipid production. Figure 2 represented response against Xylose and (NH_4_)_2_SO_4_ conc. while rest of the variables KH_2_PO_4_, Yeast extract, pH and Temperature were held constant at their centre point values (0, 0, 0, 0) i.e. 0.525 g, 0.5 g, 5 and 34 respectively. Linear surface exhibited a greater first degree effect of both independent variables on system response. An increase in sugar concentration and decrease of (NH_4_)_2_SO_4_ led to enhanced lipid production, the maximum being 1.9315 g at 30 g of the former and 0.5 g of the latter. Thus, increase in C/N ratio had a positive effect on system response which was also reported by Wiebe et al. (2012). Figure 3 depicted effect of pH and (NH_4_)_2_SO_4_ on system response. Surface was found to be more concave in this case which depicted quadratic effect of pH on lipid production. pH was found to have a positive effect on the system response and required to be maintained at high values of the same. Similar result has been reported Gong et al. (2013) where increase in lipid production has been observed by increment in initial pH. Considering both factors, maximum lipid production of 1.5424 g was obtained with pH and (NH_4_)_2_SO_4_ values of 6 and 0.5 g respectively. An effect of KH_2_PO_4_ and temperature on lipid production at fixed values of rest variables was depicted in Figure 4. It demonstrated that KH_2_PO_4_ and temperature at their maximum values of 0.70 g and 38°C led to maximum lipid production of 1.5253 g whereas temperature at its lowest value of 30°C with same KH_2_PO_4_ conc. yielded a slightly lesser value on lipid content. So the response was more sensitive to changes in KH_2_PO_4_ concentration compared to temperature when the other variables were held constant. This fact was also supported by Pareto chart diagram (Figure 1), where KH_2_PO_4_ was observed to be most significant variable amongst the selected ones. Based on the predicted model, an optimization study was carried out for maximizing lipid yield. Maximum lipid content predicted by the model was found to be 2.12 g with 30 g Xylose, 0.5 g (NH_4_)_2_SO_4_, 0.375 g KH_2_PO_4_, 0.35 g Yeast extract, pH value of 6.0 and fermentation temperature of 38°C. The data was further validated in a shake flask where the experiment was carried out under optimized condition.

### Validation of computational model

**Figure 5:**
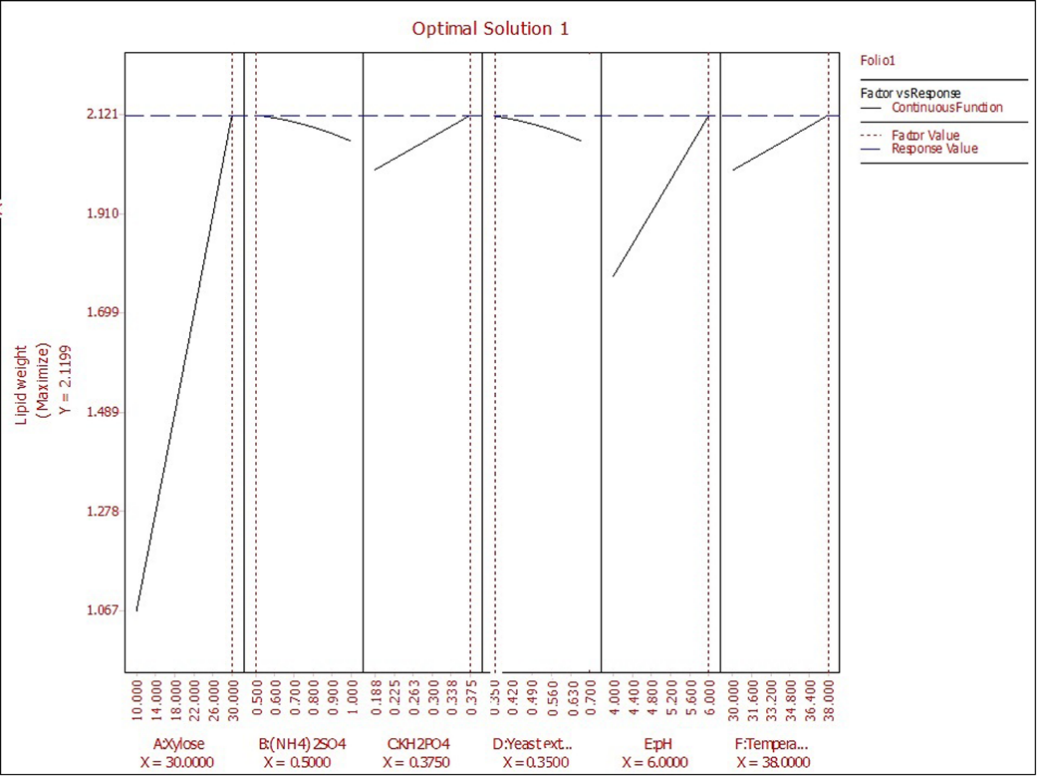
**Optimization conditions for maximizing lipid yield predicted by Reliasoft DOE.**

Validation of predicted computational model was tested in shake flask with optimized conditions yielding 2.1199 g of lipid which is almost identical to the model predicted value (Figure 5). This validated the accuracy of predicted model and confirmation of an optimum point within the system for achieving targeted lipid yield.

### Qualitative assessment of lipid

Apart from almost uniform distribution of lower carbon fatty acids (≤ C_6_ to ≥ C_14_) qualitative distribution of C_16:0_ (palmitic acid), C_16:1_ (palmitoleic acid), C_18:0_ (stearic acid), C_18:1_ (oleic acid), C_18:2_ (linoleic acid) and C_18:3_ (linolenic acid) were targeted through GC/MS. With increase in C/N ratio, lipid yield significantly increased. Irrespective of temperature, lipid yields were almost similar for C/N ratio of 25. With increase in temperature beyond 30°C, C_18:2_ (linoleic acid) was produced and C_18:3_ (linolenic acid) was not. In case of lower temperature (30°C), C_18:2_ (linoleic acid) was not present while C_18:3_ (linolenic acid) productions were observed. Almost in all 13 cases palmitoleic acid (C_16:1_) has been found to be produced which is a rare fatty acid found in microbial source. Conversion of lipid with respect to consumed carbon (C_5_ sugar) can be represented as fat coefficient (%) which was found to be nearly similar in all cases (Table 4) ranging from 6 to 8. Fat coefficient came out to be 7.06% for flask with model validation experiment which also fell within the range. It clarified the capacity of conversion of sugar into lipid by IIP-33.

## Conclusion

In this paper we have targeted to optimize maximum lipid yield on weight basis with different physical parameters and nutrient combinations as per software predicted variants. Pentose rich broth derived from lignocellulosic biomass was selected as carbon source for lipid production by IIP-33. Optimized condition was verified in shake flask which was almost identical to the model predicted value. In case of total lipid, fat coefficient are reported nearly 20 to 22% for oleaginous yeast. We have only considered non polar lipid/fatty acid fractions which would be suitable for hydro-treatment for conversion to hydrocarbon. CHCl_3_/CH_3_OH extracted total lipid which included polar lipids including phospholipids as well as glycolipids which might lead to catalyst poisoning during selective deoxygenation. n-Haxane selectively extracted non-polar fractions from CHCl_3_/CH_3_OH extractives which lowered the fat coefficients by ~50%, but optimization on this basis would definitely help to further scale up lipid production from cheap biomass source like sugarcane bagasse.
